# Domestic laundry and microfiber pollution: Exploring fiber shedding from consumer apparel textiles

**DOI:** 10.1371/journal.pone.0250346

**Published:** 2021-07-09

**Authors:** Ekaterina Vassilenko, Mathew Watkins, Stephen Chastain, Joel Mertens, Anna M. Posacka, Shreyas Patankar, Peter S. Ross

**Affiliations:** 1 Ocean Wise Conservation Association, West Vancouver, British Columbia, Canada; 2 Sustainable Apparel Coalition, San Francisco, California, United States of America; VIT University, INDIA

## Abstract

Synthetic fibers are increasingly seen to dominate microplastic pollution profiles in aquatic environments, with evidence pointing to textiles as a potentially important source. However, the loss of microfibers from textiles during laundry is poorly understood. We evaluated microfiber release from a variety of synthetic and natural consumer apparel textile samples (*n* = 37), with different material types, constructions, and treatments during five consecutive domestic laundry cycles. Microfiber loss ranged from 9.6 mg to 1,240 mg kg^-1^ of textile per wash, or an estimated 8,809 to > 6,877,000 microfibers. Mechanically-treated polyester samples, dominated by fleeces and jerseys, released six times more microfibers (161 ± 173 mg kg^-1^ per wash) than did nylon samples with woven construction and filamentous yarns (27 ± 14 mg kg^-1^ per wash). Fiber shedding was positively correlated with fabric thickness for nylon and polyester. Interestingly, cotton and wool textiles also shed large amounts of microfibers (165 ± 44 mg kg^-1^ per wash). The similarity between the average width of textile fibers here (12.4 ± 4.5 μm) and those found in ocean samples provides support for the notion that home laundry is an important source of microfiber pollution. Evaluation of two marketed laundry lint traps provided insight into intervention options for the home, with retention of up to 90% for polyester fibers and 46% for nylon fibers. Our observation of a > 850-fold difference in the number of microfibers lost between low and high shedding textiles illustrates the strong potential for intervention, including more sustainable clothing design.

## Introduction

Plastic microfibers are considered one of the dominant forms of microplastic pollution (particles < 5 mm) in the world’s oceans [1, 2]. While the ecological impacts of microplastics remain unclear, ingestion by a number of aquatic organisms has been readily demonstrated in both laboratory and real world studies [3–6]. Adverse effects may include entanglement of feeding appendages, gut blockage and malnutrition in zooplankton or lower levels of the food web [3, 6–9].

The potential vulnerability of zooplankton populations underscores concerns about the implications of microplastics for food web health and ocean productivity [7]. As opposed to fragments and other more regularly shaped microplastics, fibers may cause entanglement or blockage in the gastrointestinal tracts of filter feeders due to their elongated shape. With very long residence times in the environment (e.g. 58–1200 years, [10]) microplastic pollution represents an important subject of scientific and policy concern.

The origins of microfibers found in aquatic environments are increasingly linked to the domestic laundry of textiles, with early global estimates indicating that households contribute up to 35% of microplastics discharged to the environment (e.g. microbeads in cosmetic and personal care products, microfibers originating from synthetic textiles, tire particles, microplastics originating from the application of paint) [11, 12]. Synthetic fiber polymers found in the marine environment include polyester, acrylic, polypropylene and nylon [12], all representing common constituents of clothing. Textile laundering has been shown to release between 120 and 730,000 microfibers into domestic wastewater per cycle, or up to 0.1% of textile mass every wash [12–17]. Synthetic fibers are common in treated municipal wastewater effluent [18, 19] and have been documented in urbanized areas near wastewater treatment facilities [12, 20].

A few studies have provided basic insights into the interplay of textile design and laundry practices as they influence fiber loss. Factors shown to influence fiber shedding during home laundry include the type of washing machine used, age of fabrics washed [15], water temperature [17, 21], type of detergent [13], wash speed [22], textile construction [14, 16, 17, 23, 24] and chemical finishing [25]. Knitted yarn constructions have been reported to have both higher [23] and lower fiber shedding compared to woven yarn geometries [24]. Challenges in documenting and characterising microfiber loss from textiles are partly due to differences in assessment methods used, the wide variety of materials and products on the market, and the often confounding interplay among factors that contribute to shedding during laundry.

The goal of this study was to characterise the loss of fibers from a variety of synthetic and natural consumer fabrics during home laundry. We explore here a variety of material types, constructions and treatments used in textile manufacture, with the objective of characterizing the factors shaping microfiber loss and informing on this source of microfibers to the aquatic environment. In addition, we evaluated the efficacy of two commercially-available washing machine lint traps (*Lint LUV-R*^*©*^ and *Filtrol*^*©*^).

## Methods

### Textile sample characteristics and preparation

We established a novel ‘Microfiber Partnership’ with outdoor retailers (MEC, Patagonia, Arc’teryx and REI), a wastewater treatment plant authority (MetroVancouver) and the Canadian government (Environment and Climate Change Canada) to inform the design and to carry out this study of textile shedding. Thirty-seven textile samples (*n* = 37) were provided by members of our Microfiber Partnership to evaluate fiber loss during laundry (Table 1; S1 Table in S1 File).

**Table 1:** A summary description of textiles assessed for fiber loss during laundry, with the number of material types evaluated.

Class of textiles	Description	Total (*n*)	Polymer composition
Fleece	Knit textiles made with synthetic filament yarn and with raised and trimmed nap	10	100% polyester: virgin (*n* = 6), recycled (*n* = 4)
Jersey (mechanically treated)	Knit textiles made with filament yarn and mechanically treated (sanded, brushed or piled)	5	100% polyester: virgin (*n* = 3), recycled (*n* = 1), 100% nylon (*n* = 2)
Knit filament	Knit textiles made with filament yarn, not mechanically treated	2	100% polyester virgin (*n* = 1), nylon-polyester blend (*n* = 1)
Knit spun staple	Jersey with no mechanical treatment (*n* = 3), cotton-faced fleece (*n* = 1)	4	100% cotton (*n* = 1), cotton-polyester blend (*n* = 2), 100% wool (*n* = 1)
Woven filament	Woven textiles made with filament yarn: no mechanical treatment (*n* = 6), brushed (*n* = 1), crinkled (*n* = 1)	8	100% nylon virgin (*n* = 6), nylon recycled (*n* = 1), polyester recycled (*n* = 1)
Woven spun staple	Woven textiles made with spun staple yarn and brushed	3	100% cotton (*n* = 2), polyester virgin (*n* = 1)
Composite	Woven textiles made with filament yarn insulated or covered with non-fibrous polymer	5	Nylon-ePTFE (*n* = 2), nylon-polyester composite (*n* = 2), polyester-PU (*n* = 1)

ePTFE: polytetrafluoroethylene; PU: polyurethane.

Case series study design was used as a screening tool for the generation of hypotheses regarding the effect of textile design on the shedding [26]. The textiles tested varied in polymer composition (cotton, wool, polyester, and nylon, in some cases mixed with spandex or elastane), yarn type (spun-staple or filament), textile construction (knit or woven), mechanical treatment (brushed, sanded or sheared), and chemical finishes (anti-pilling, softener, wicking, Durable Water Repellent [DWR], and/or anti-odour). Before testing, each fabric was cut into 66 cm x 66 cm swatches with double-hemmed edges. Load weight for each test was standardized to 500 g. A smaller hemmed swatch was also used if required for incremental weight adjustments.

### Laundering conditions and effluent processing

We designed and established a dedicated ‘washing machine research center’ to conduct our study. Three top-loading laundry machines (SDL Atlas Vortex M6, Rock Hill, South Carolina, USA) were installed with custom-designed sampling manifolds connected to the machine drain hoses (S1 Fig in S1 File). The machines and sampling equipment were enclosed in a dust-protective tent made from non-shedding (recyclable) polyethylene sheets and supported by a frame made of polyvinyl chloride (PVC) pipes. To further reduce airborne contamination during laundry experiments, ion-generating air filters (Honeywell HFD-010C) were operated inside the tent during the laundry tests. All surfaces were regularly cleaned. Water for all laundry cycles was filtered with 5 μm pore-size filters. While particles smaller than 5 μm could have been missed with this pore size, the diameters of textile fiber (> 10 μm), their generally elongate lengths, and the limitations of the human microscope observer all point to this size limit being appropriate for rigorous enumeration.

Sampling manifolds were designed and constructed for the continuous collection of laundry effluent sub-samples during the entire laundry cycle (S1 Fig in S1 File). The purpose of this sampling approach was to balance the need to account for the variation in fiber shedding between different cycles and attain reliable quantification of microfibers by count and mass. The manifolds consisted of three levels of tubing splits, with decreasing sizes of tubing selected. Through an iterative approach, optimal hose diameters were chosen to accommodate the drop of water volume after a split, while minimizing turbulence and backpressure. The final internal diameters of the tubing were 3/4”, 5/8” and 3/8”, respectively. The laundry procedure was adapted from the AATCC 135–2004 method for an average domestic laundry cycle, with conditions detailed in S2 Table in S1 File [27].

Before each test wash, two wash cycles were run without samples to minimize possible background contamination. A standardized textile sample (500 g) was then washed five times, and a sample from the laundry effluent was continuously collected during each laundering cycle. For most textiles, 1/8 of the total effluent from a single cycle was collected (10 L), except in the case of five materials that had low shedding rates where the volume was increased to 1/4 (20 L) to enable adequate gravimetric analysis. The effluent was collected into a sealed polypropylene bucket (S1 Fig in S1 File). The machines were cleaned using a shortened high-volume wash cycle at least two times between each sample wash. Effluent samples were then vacuum-filtered through 20 μm polycarbonate filters (45 mm diameter) under a laminar flow hood with filtered air. The polycarbonate filters, with captured microfibers from laundry effluent runs, were dried in closed Petri dishes at 50°C overnight and then stored in individually sealed bags containing desiccant pouches. They were then weighed immediately after removal from the desiccator bags.

Duplicates were collected from a subset of samples (*n* = 18, high and low shedding) to determine method precision. The coefficient of variation ranged from 0 to 27.6%, with an average of 11.5%. Procedural blanks were collected every six washes, in parallel with the test washes. A procedural blank sample was collected for laundry washes performed without a textile with the resulting effluent processed in the same manner as a test sample. The total number of fibers calculated for the total blank effluent volume (1/8) varied from 300 to 3,670, with an average 1,808 ± 914, which accounted for <1%– 14% of the total fibers released by textiles during the wash. As can be expected, a higher percentage of background-to-sample fibers was detected for the lowest-shedding fabrics. Weights from the procedural blanks are not reported and were not used for sample weight correction because they were too low for accurate gravimetric analysis.

Spectral analysis using Fourier Transform Infra Red Spectrometry (FTIR) of textile samples prior to laundry testing enabled a critical evaluation of results following wash cycles, with no unexpected contamination of the laundry testing system with any plastic materials used in the collection apparatus (e.g. polypropylene or polycarbonate). The only exception in this case was the observation of large numbers of contaminating mystery particles from a subset of textile samples laundered, which we describe in the Results and discussion.

### Lint quantification

The average lint mass from wash cycles 3–5 was used to compare shedding among textiles since we found first wash cycles often released higher amounts of fibers in preliminary experiments.

In addition to textile fiber mass, we estimated fiber numbers released by each sample textile. Precise counts were not possible because of exceedingly high numbers, and the challenges associated with clumping of fibers masses on filters. Therefore, for each textile, a subsample was selected where both mass and fiber count could be reasonably evaluated. Fiber counting was performed visually using a stereomicroscope equipped with 18.4x maximum magnification, and a camera connected to Olympus DP2-SAL firmware analysis software. A coefficient *k* was calculated for each textile as the ratio of lint mass to fiber count.

The reproducibility of this approach was evaluated for six textiles by calculating the coefficient *k* twice from subsamples from two separate washes. The relative standard deviation of *k* varied from 0.3% to 55% with an average of 25%, indicating that the calculated fiber counts have relatively low precision. In several cases, enumeration of fibers was not possible due to fiber entanglement. Moreover, the presence of non-fibrous fragments or residue observed in some of our lint samples likely affected coefficient *k* by increasing the lint weight. In such cases, no fiber count was reported for the tested textile. Due to high counting errors and an incomplete data set, fiber numbers were not used for any statistical analysis and serve as an estimate of fiber textile footprint by number.

### Fourier Transform Infrared Spectroscopy (FTIR) analysis

Fourier-transform infrared spectroscopy was used to confirm the polymeric composition of textiles and to identify the unexpected non-fibrous materials released by several samples. Microfibers and additional materials lost during a laundry cycle test were analyzed using a Cary 670 FTIR spectrometer equipped with a Cary 620 microscope (Agilent Technologies, Mulgrave, AUS). Particles larger than 3 mm, including fragments of textiles, were analyzed with the bench-top attenuated total reflectance (ATR) accessory equipped with a MIRacle Diamond / ZnSe crystal (Pike Technologies, Madison, USA). Particles smaller than 3 mm, including individual microfibers, were analyzed using the micro-ATR accessory equipped with a Germanium crystal [18].

The smaller particles were manually affixed to a glass microscope slide covered with a thin layer of 2% dextrose (Sigma-Aldrich, St Louis, USA) as an adhesive agent to enable FTIR analysis. For each background and sample, 128 co-added scans were collected at a resolution of 8 cm^−1^ in the range of 3,800 to 900 cm^−1^. FTIR imaging spectra were matched against a commercial polymer database with 250,000 entries (*KnowItAll*, Bio-Rad) of selected ATR-FTIR spectra that included polymers, plastics, polymer additives, plasticizers and packaging materials. Sample spectra were deemed to be positively identified if i) all major peaks were present in a sample and in reference spectra, and ii) the total overlap of the sample and reference spectra was greater than 80% [28].

### Lint trap fiber efficiency evaluation

Two commercially available washing machine lint trap models, Filtrol^©^ (WEXCO, Milaca, MN, USA) and Lint LUV-R^©^ (Environmental Enhancements, Dartmouth, NS, Canada) were evaluated for their ability to remove textile fibers shed during home laundry. Fiber capture was quantified for three filter pore sizes in case of the Filtrol^©^ model (50 μm, 100 μm and 200 μm), and two pore sizes for the Lint LUV-R^©^ model (150 μm and 1588 μm). Both filter models were tested using two types of textiles: i) polyester fleece and ii) nylon stretch woven. Replicate washes for both fabrics were run with all mesh sizes, except the two largest meshes (200 μm and 1588 μm) for which nylon fiber retention was not assessed.

Laundry effluent downstream of the lint filter (the lint-filter filtrate) was passed through a 10-μm stainless-steel conical filter (S2 Fig in S1 File). The lint retained by the candle filter (a cylindrical Hop Randall filter with a height of 33 cm and diameter of 12.7 cm with 10 um stainless steel cartridge was used) and the lint captured by the lint filter was then separately collected using the 20-μm vacuum-filtration and weight as described in section 2.3. Lint retention was calculated for each combination of mesh size and textile type as a weight/weight percentage of lint retained by the lint filter versus the total mass of shed lint, both recovered from the lint filter and the filter filtrate, as below:

$$\%\;mass\;retained=\frac{solids\;in\;filter}{solids\;in\;filter+solids\;in\;the\;filtrate}$$

Additional tests were run to compare the two sampling methods: (1) the candle-filter method for processing the entire effluent, used for evaluating lint filters, and (2) the manifold method for sampling 1/8 of the laundry effluent, used for evaluating textile shedding. For three washes, 1/8 of the laundry effluent was collected using the manifold, and the remaining 7/8 were processed with the candle filter. The two samples from each wash were used to make separate calculations of the total lint from the wash, and the results were compared. The lint mass calculated from using the candle filter was on average 14% lower compared to the mass calculated using the manifold, indicating that sampling method is yet another source of variability potentially affecting inter-comparability of textile shedding studies.

### Estimates of fiber emission by Canadian and U.S. households

We estimated the extent of microfiber shedding during laundry by households in Canada and the U.S.A. using the average microfiber loss by weight (131 mg per kg of textile per wash) and count (514,400 microfibers per kg of textile per wash) by all synthetic fabrics in this study. Data was converted using the average weight of a domestic laundry load (4 kg). Natural fabrics (i.e. cotton and wool), as well as blends containing natural fibers, were excluded from this estimate.

The annual total household release of synthetic microfibers from laundry in Canada was estimated using a total of 14,072,080 households [29], and an average of 218 laundry loads per year per household [30]. For the U.S.A., annual synthetic microfiber release from laundry was estimated using a value of 127,586,000 households [31] and an assumed 300 laundry loads per household per year [32]. To estimate microfiber emission via municipal wastewater, we assumed a 95% retention in facilities based on data from studies of primary, secondary, and tertiary wastewater treatment plants [18, 19]. These estimates consider the proportion of domestic wastewater that is untreated or collected into household septic tanks in Canada [33] and the U.S.A. [34].

### Data analysis

All data were blank-corrected (mass and counts). All statistical analyses were performed using the R software environment (R Core Team, 2015) using data from 37 samples. To assess the independence of textile parameters, we applied ANOVA (in case of a continuous and categorical variable) or Chi-squared Test of Independence (in case of two categorical variables). All tests in this study were performed on Ln transformed data as fiber shedding dataset displayed non-normal distribution. Multi-comparisons between major textile groups (polyester, nylon, natural) were carried out using a non-parametric Kruskal–Wallis test with a post hoc Dunn’s test. Shedding values for each category are presented as mean ± Standard Deviation unless stated otherwise. Further, a two-sample t-test was applied in a few cases to explore the effects of material parameters within these categories.

In addition, we used a machine learning Random Forest (RF) method in R as a complementary technique to examine the relative influences of textile properties on fiber shedding [35–37]. The RF method is a supervised machine learning method which involves producing multiple regression trees, which, in turn, are then combined to make a single consensus prediction for a given observation [37]. We applied the RF model to explore the role of textile parameters in fiber shedding using the *randomForest* R package [36]. The mean squared error (abbreviated MSE) represents a measure of the accuracy of the RF model, where as the IncNodePurity (mean decrease in Gini score) is a measure of variable importance and can be used to rank variables by the strength of their relation to the response variable. The higher the value of MSE and IncNodePurity the higher the importance of variables in the model. All data are presented as mean ± Standard Deviation unless otherwise stated.

The fiber shedding rates, underlying figures and statistical analysis are available in S1 File.

## Results and discussion

**Fig 1:**
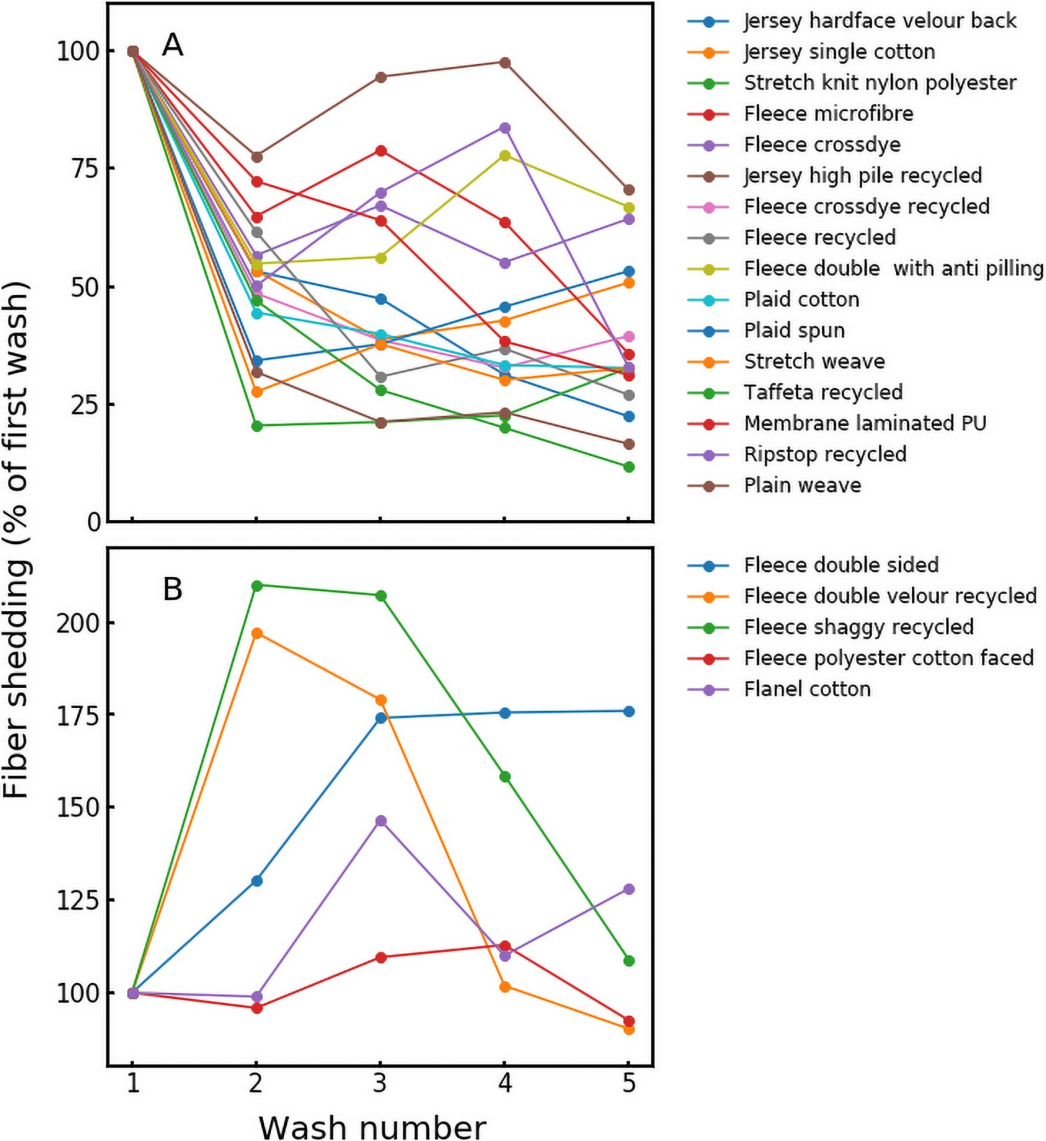
Textile fiber loss varied over consecutive laundry cycles, with some textiles losing less fibers with subsequent wash cycles (A), and some losing more or exhibiting a relatively steady release (B). This underscores the potential for the use of industrial pre-wash procedures to reduce microfiber pollution. Data is presented as a percent (%) of lint mass released in the initial wash for 21 select materials.

Textile fiber shedding varied widely among samples tested, ranging from 9.6 to 1,240 mg of lint kg^-1^ of textile washed, or an estimated 8,809 to > 6,876,000 microfibers (Fig 1; S1 Table in S1 File). Shedding footprints of synthetic textiles were generally higher than those observed elsewhere [12, 17, 23, 38, 39], although a direct comparison must be carried out with caution given the diversity of methods used (S3 Table in S1 File).

Several textile parameters were non-independent in our study (construction, fiber material and mechanical treatment; Chi-squared Test of Independence, *p*<0.04), constraining a causal determination of the specific factors explaining fiber loss among different samples. For this reason, we focussed our analysis on comparing fiber shedding between pure polyester, nylon, and natural-based textile samples, with an additional examination of design influences within these categories.

### Fiber release varied with consecutive washing

Initial laundry cycles in our study generally exhibited higher fiber losses than subsequent cycles (Fig 1A), consistent with recent reports [17, 23, 39]. We hypothesize that higher initial wash shedding was due to the presence of loose fibers and particles from the manufacturing process. These observations point to opportunities for reducing consumer-end particle release by implementing a wash step for new materials at manufacturing facilities. However, findings did vary among products tested, with some materials releasing more fibers with subsequent laundry cycles, while others exhibited a relatively steady release (Fig 1B).

### Fiber shedding in laundry varied among textiles

**Fig 2:**
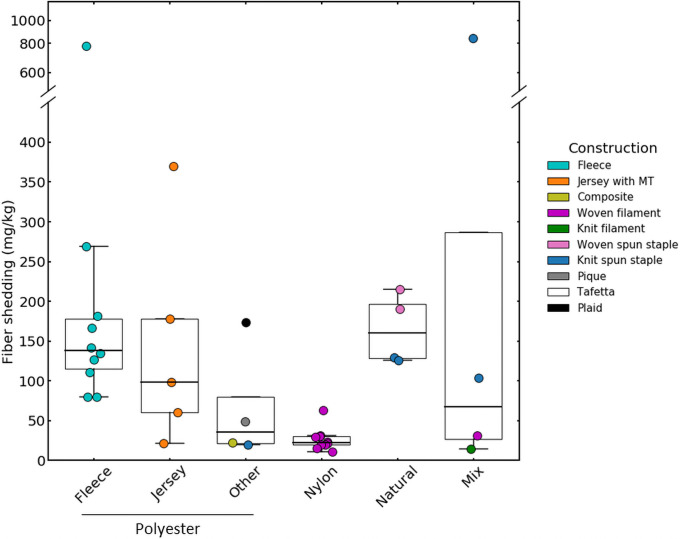
Fiber shedding varied across major textile categories. On each box plot, the central mark indicates the median, and the box extends to the 25^th^ and 75^th^ percentiles, respectively. Data points overlaying box plots represents an average of lint mass per kg of textile per was (mg) from last three washes for individual fabrics.

The complex interplay among factors thought to be influencing fiber loss among textile samples rendered it exceedingly difficult to identify the dominant factors contributing to shedding, but some patterns emerged from our study. Fiber shedding was generally higher in the polyester (mean ± SD = 161 ± 173 mg kg^-1^ of textile washed, *n* = 18) and natural (165 ± 44 mg kg^-1^ of textile washed, *n* = 4) material categories, in contrast to nylon materials which had ~ six fold lower fiber shedding masses (27 ± 14 mg kg^-1^ of textile washed, *n* = 9). Textiles made from polyester were diverse in terms of design, and included those with recycled fibers, differing mechanical treatments, knit and woven construction, filamentous yarns, and composite designs (Fig 2; S1 Table in S1 File). However, the polyester category was dominated by mechanically-treated fleeces (*n* = 10) and jerseys (*n* = 5) with filamentous yarns, materials that have been previously noted for their high fiber losses [23, 40]. Polyester is an important material used in the global textile sector [41], highlighting the importance of identifying the design elements that trigger shedding during the lifetime of a product.

No differences were found in fiber shedding between polyester fleeces and jerseys (Fig 2, Student’s t-test, *p* = 0.3), with both groups of textiles displaying a wide range of fiber loss. Interestingly, while polyester fleece designs fared worst with regards to fiber loss during laundry [23], there were also some low shedding fleeces (Fig 2, S1 Table in S1 File). This suggests the need to carefully scrutinise oversimplified strategies for textile design choices, as some have suggested fleece avoidance as one mitigation opportunity for textile-related microfiber pollution [42]. Of note is the low fiber shedding of the few non-mechanically treated materials examined in our study (30 ± 16.1 mg kg^-1^ of textile per wash, *n* = 3, S1 Table in S1 File), which was lower than that of mechanically-treated fleeces (204 ± 198 mg kg^-1^ of textile per wash, *n* = 10, Kruskal-Wallis test with post hoc Dunn test, *p*<0.05).

In addition to univariate approaches to characterising the factors shaping fiber loss, our application of a Random Forest model strengthened our identification of the primary factors driving fiber loss. In this way, mechanical treatment, material type and material density were identified as the three leading factors influencing fiber shedding in our Random Forest model (performed on all textiles (*n* = 37), S3 Fig in S1 File).

Textiles made with nylon shed less (mean of 27 ± 14 mg kg^-1^ of textile per wash) than those made with polyester (mean of 161 ± 173 mg kg^-1^ of textile per wash; Student’s t-test *p*<0.05). All nylon textiles were constructed with woven filament yarns, had composite or non-composite designs, and released similar fiber quantities to polyesters of the same construction, suggesting that properties other than polymer type influenced shedding.

The few natural materials examined here also shed considerable amounts of fibers, which may be due to their inherent short spun-staple yarn construction. These samples released similar quantities (100% cotton and wool, 165 ± 44 mg kg^-1^ of textile per wash, *n* = 4) to the polyester samples (161 ± 173 mg kg^-1^ of textile per wash, *n* = 18). Higher fiber losses from cotton compared to polyester textiles during laundry has been reported in two recent studies [17, 43]. Others have attributed the differences to the fuzz formation tendencies of polyester and cotton fibers, with high yarn hairiness and low yarn breaking strength for cotton fibers [17]. Although natural fibers are subject to microbial degradation in wastewater treatment plants and the environment [17], their recent detection in remote ocean compartments [44, 45] highlights the need for additional research into the ultimate distribution and fate of natural fibers in the environment.

Mixed polymer fiber samples were characterized by variable shedding. The lowest values observed in our study were in the two nylon-polyester filament yarn composites (46%/46% with 8% elastane; and 35%/49% with 16% elastane, S1 Table in S1 File). While both differed in construction (woven vs knit), they were not mechanically treated, which may help explain their low shedding. The single mechanically-treated cotton-polyester fleece (50%/50%) material emitted some of the highest quantities of fibers (838 mg kg^-1^ of textile per wash), whereas the non-mechanically-treated cotton-polyester (50%/50%) jersey exhibited intermediate shedding (103 mg kg^-1^ of textile per wash).

**Fig 3:**
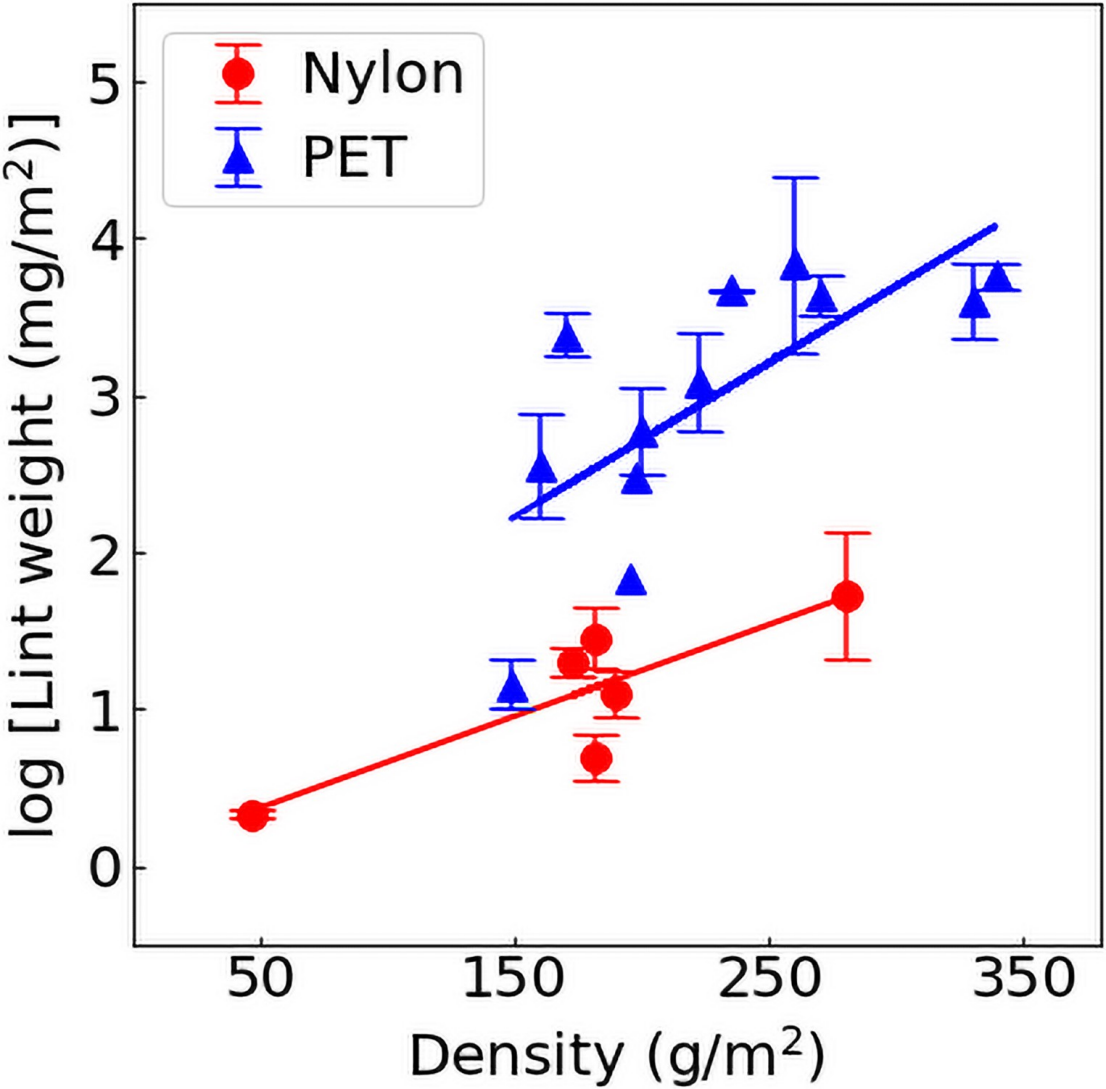
Fiber shedding in synthetic materials was positively correlated with fabric area density. Least squares regression was performed on fiber shedding mass (Log-transformed) and sample material density (weight per m^2^) of nylon (*n* = 8) and polyester (*n* = 11) textiles. Polyester: y = 0.011x + 0.473 R^2^ = 0.55, *p* = 0.007; R^2^ = 0.55, *p* = 0.007; Nylon: y = 0.0058x + 0.0497, R^2^ = 0.7641, *p* = 0.023.

Fiber shedding from synthetic textiles was influenced by material area density. Shedding from both nylon and polyester materials, including samples made of 100% pure fiber and those with up to 12% elastane, correlated positively with sample material weight per square meter (g/m^2^; Fig 3). This suggests that textile shedding occurs not only from the surface of the textile, but also from deeper layers, consistent with observations elsewhere [23]. Further study in this regard is needed, though, as others have found no influence of material area density on fiber loss during laundry [24].

### Properties of microplastics shed from textiles

**Fig 4:**
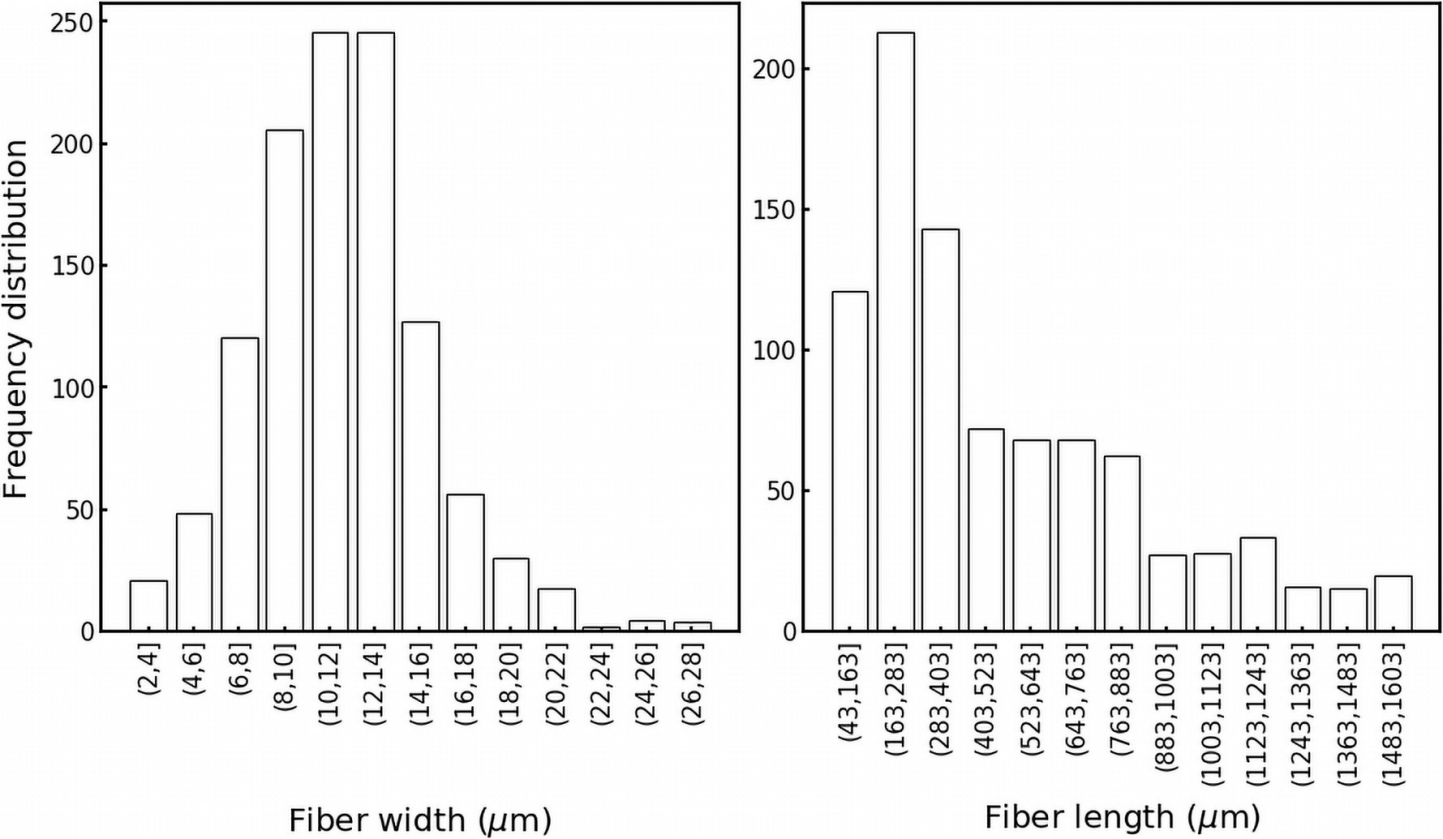
Fiber lengths and widths displayed different frequency distribution profiles. Frequency distribution was obtained for all 37 materials characterised in the study using average fiber dimensions from each laundry effluent sample. Fiber width displayed a symmetrical distribution, with a mean value of 12.4 μm (bin width 2 μm). Fiber length was skewed to the right, with a median length of 405 μm (bin width = 150 μm). Note, outliers beyond the minimum and maximum values here (40–2,240 μm) are not included in this graph.

The median width of fibers for each textile was 12.4 ± 4.5 μm (range 2 to 37 μm), and the median length was 405 ± 1,086 μm (range 48 to 10,272 μm; Fig 4, S1 Table in S1 File). Interestingly, the width of fibers from all fabrics varied over a narrow range and had a frequency distribution that was nearly symmetrical around the median (Fig 4A). In contrast, the length distribution was heavily skewed to the right (Fig 4B), suggesting that fibers typically break lengthwise rather than widthwise.

Fiber width and length did not differ among major textile categories (polyester, nylon and natural) and did not correlate with shedding masses, except for nylon (S4 Fig in S1 File). The length and width of shed fibers did not differ among sub-categories including cotton, wool, virgin polyester, recycled polyester, and virgin nylon. However, recycled nylon released longer fibers than all other materials (ANOVA, Dunn’s post hoc, *p*<0.05). This indicates that fiber breakage is complex, and that breakage may be unrelated to fiber length.

Fiber dimensions fall into the range of those reported in other studies of laundry [15, 24], municipal and textile industry wastewater treatment facilities [19, 46], and the aquatic environment [47]. There is also an overlap between the dimensions of textile fibers here and those ingested by aquatic organisms in laboratory studies and in the natural environment. Polyester fibers of 300 μm in length were readily available to freshwater crustacean *Daphnia magna*, but even larger fibers of 1,400 μm were detected in some individuals [8]. Wild zooplankton from the NE Pacific Ocean consumed microfibers in the range of 461–1,778 μm [3]. Shorter fibers may more readily transit washing machines and lint traps (e.g. [21]), such that they are more likely to be released into the receiving environment and made available for ingestion by biota.

**Fig 5:**
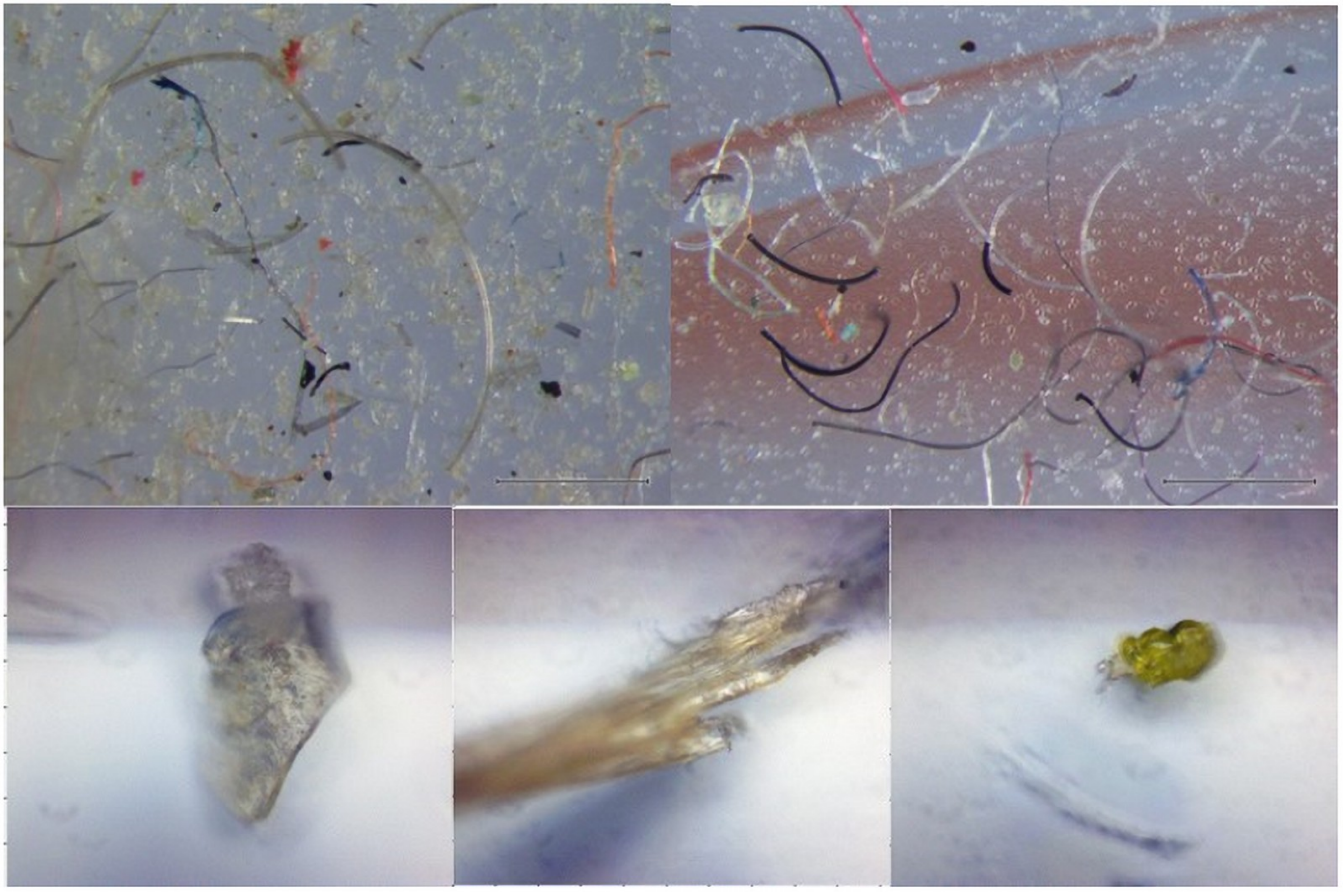
Non-fibrous particles were released by several textile samples during laundry testing (*n* = 6). Top left panel: example of the filter containing mixture of fibers and non-fibrous residue; top right panel: an example of a filter with mostly fibers and no notable non-fibrous residue. Bottom panel: examples of non-fibrous particles.

In addition to the loss of microfibers from new textile samples during laundry, we found surprisingly large amounts of non-fibrous fragments released by several samples (*n* = 6, Fig 5). FTIR analysis revealed these as comprising a variety of natural materials and plastic polymers including acrylic co-polymer, polyesters (including polyethylene terephthalate), polystyrene, aminoplast resin, vinyl acetate co-polymer and cellulose. Chemical finishing may partly explain these fragments, but we found no relationship between the presence of these particles and reported chemical finishing for these products (Chi-Square test, *p* = 0.35). This suggests that there may have been non-reported chemical components or materials in the samples we evaluated. These observations suggest that textiles may be a source of microparticles to the wastewater stream other than just those deliberately used in the design and manufacture of the product.

### North American release of microfibers from laundry

The extrapolation of synthetic textile fibers shed from this study (131 mg kg^-1^ of textile per wash) enabled an estimate of annual fiber emissions by households in Canada and the U.S.A. This assessment underscores the potential of textile design and domestic intervention as an important area to target for fiber pollution reduction [11, 48]. In this way, we estimated the average household to generate up to 135 g of plastic microfibers annually (or estimated 5.33 x 10^6^ microfibers), which amounts to 22 ktonnes of fibers (or 85 x 10^15^ microfibers) entering WWTPs in Canada and the U.S.A. combined.

After treatment, the cumulative fiber emission to the aquatic environment (streams, lakes, estuaries, and oceans) in Canada and the U.S.A is estimated at 878 tonnes (or 3.5 x 10^15^ microfibers). In addition, significant amounts of fibers appear in municipal sludge (4 ktonnes, or 16 x 10^15^ microfibers), which depending on municipal practices, may end up in agricultural or other terrestrial applications [49]. Our estimates illustrate the important retention of microplastics in WWTP, with 63–80% ending up in sludge and biosolids [18, 50, 51]. The latter presents an as-yet under-characterised pathway for microplastics to the wider environment.

**Fig 6:**
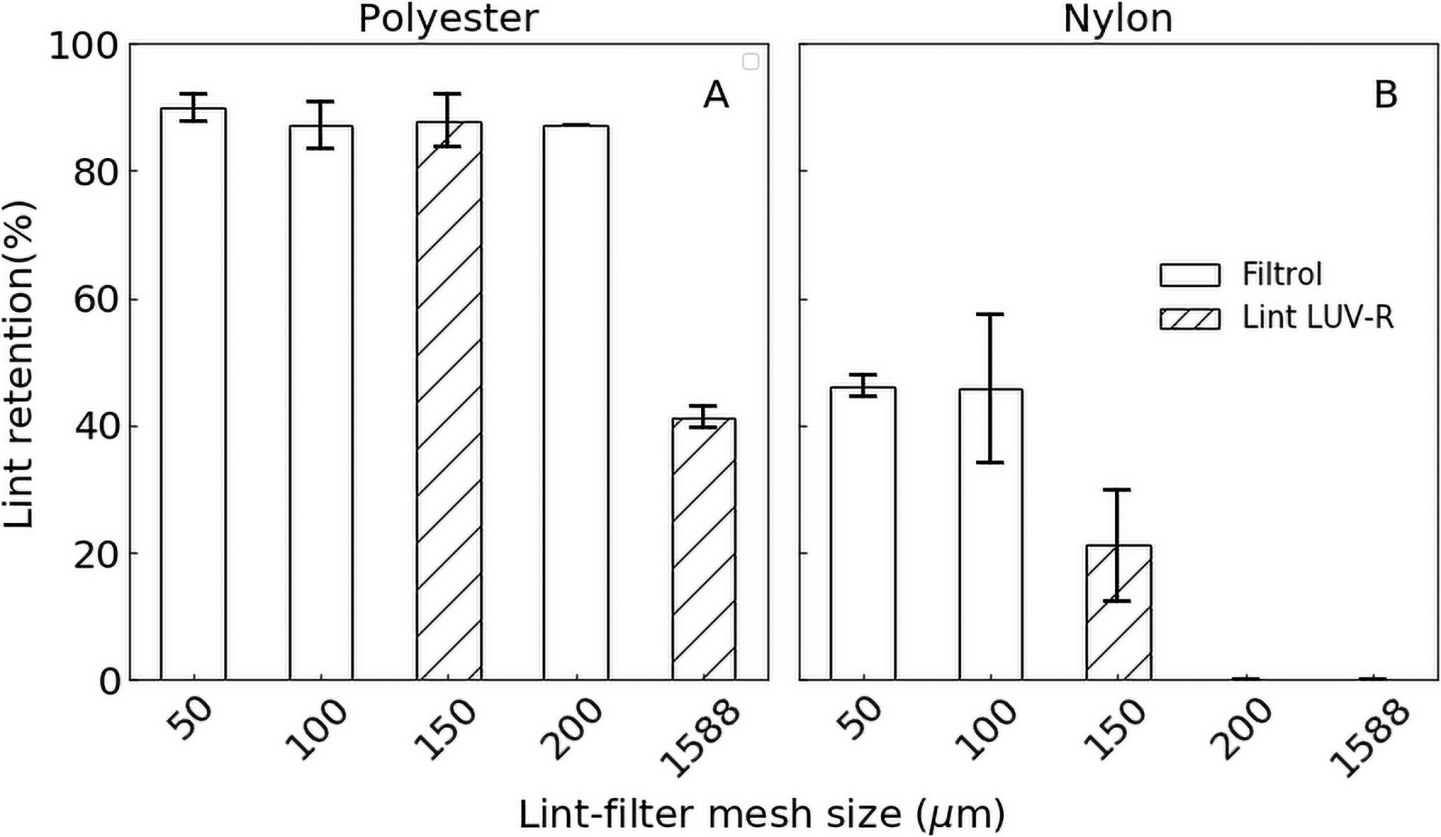
Washing machine lint trap retention differed between textile type and lint trap mesh size. Polyester was retained to a greater degree (A) than nylon (B) in lint traps. Error bars show 1 standard deviation around the mean. No error bar is shown when *n* = 1. N.D.–not determined.

Aftermarket washing machine lint traps are available to the consumer to reduce textile fiber emissions from homes. Only one other study has thus far investigated the effectiveness of such a device (Lint LUV R, [48]). We found lint traps to offer benefits in terms of textile fiber reduction, but their success depends on the material type and device mesh porosity. Internal mesh porosities of 50–200 μm captured between 88.2 and 89.9% of polyester fibers by weight (Fig 6; consistent with the other report [48]), despite the width of these fibers being much smaller than mesh pores (median in this study 12.4 ± 4.5 μm). However, the retention of polyester fibers decreased to 41.2% with the largest mesh porosity of 1,588 μm. In contrast, only 46.0 of nylon fibers were retained by 50 μm, and 100 μm meshes and the retention dropped further down to 19.4% with a 150 μm mesh. The retention difference may be at least in part caused by high dimensional stability of polyester fibers [52] and a likely stronger resistance to flow-through in the lint filters.

Textile design improvements provide an additional opportunity to reduce fiber pollution in the environment. Research in this area is relatively new and constrained by a lack of consistent testing methods and standards (as reviewed in [53]), which limits the ability to fully understand the emissions and risks of textile fibers in aquatic environments. Concerted efforts are therefore needed to develop practices that enable comparisons in research and, most importantly, environment monitoring [23, 54].

Microfiber pollution adds to an established list of environmental impacts associated with textile manufacture, trade, sale, use and disposal. There exists an opportunity to strengthen best practices in the textile sector through material life-cycle impact analysis (e.g. Higgs Sustainability Materials Index or *MADE-BY* Fiber Benchmark [55]). Textile production (synthetic and natural) is estimated to produce 1.2 billion tonnes of CO_2_ [56], and additional environmental costs include water consumption, and the use of chemicals including flame retardants and waterproofing agents [53, 55]. Environmental footprints vary among the different textile types manufactured, with sustainability requiring a concerted evaluation of all impacts pertaining to the life cycle of products, from design to manufacturing, use and end of life. In this regard, fiber shedding represents an emerging concern–and opportunity for mitigation–for the textile, waste management and environmental management sectors.

## Supporting information

S1 File (DOCX)
