# Supplementary material for: Domestic laundry and microfiber pollution: Exploring fiber shedding from consumer apparel textiles
Source: PLoS One. 2021 Jul 9;16(7):e0250346. doi: 10.1371/journal.pone.0250346 (PMC8270180; doi:10.1371/journal.pone.0250346)
Supplement: S1 File — (DOCX) [file pone.0250346.s001.docx]

S1 File for

**Domestic laundry and microfiber pollution: exploring fiber shedding from consumer apparel textiles**

Ekaterina Vassilenko^1^, Mathew Watkins^1^, Stephen Chastain^1^, Joel Mertens^2^, Anna M. Posacka^1^, Shreyas Patankar^1^, Peter S. Ross^1*^

^1^ Ocean Wise Conservation Association, 4160 Marine Drive, West Vancouver, BC Canada V7H 1H2

^2^ Sustainable Apparel Coalition, PO Box 237, San Francisco, CA USA 94104-0237

Corresponding author: [pross@eoas.ubc.ca](mailto:pross@eoas.ubc.ca)

**S1 Table**. Fiber shedding rates of 38 textiles investigated in this study. Data is presented as the average weight of lint and an estimated number of fibers of the last three laundry washes (average ± standard deviation [SD]). Fiber length and width medians are based on the analysis of at least 20 fibers. MT – mechanical treatment. ND – not determined. * Type of mechanical treatment was not reported.

| **Construction** | **Yarn type** | **Product**  **description** | **Fiber polymer composition** | **MT** | **Fiber shedding** | | **Fiber length** | | **Fiber width** | **Textile**  **classes** |
| --- | --- | --- | --- | --- | --- | --- | --- | --- | --- | --- |
|  |  |  |  |  | **Mass, Average ± SD**  **(mg per kg of textile per wash)** | **Count, Average ± SD**  **(MF per kg of textile**  **per wash)** | **Median ± SD (µm)** | | **Median ± SD (µm)** |  |
| Knit | Spun staple | Heather single jersey | 100% cotton | None | 126 ± 18 | 764,406 ± 106,667 | 669.5 ± 318.2 | | 11.7 ± 1.2 | Knit spun staple (n=4) |
|  |  | Cotton-faced fleece | 50% cotton,  50% polyester | Napped | 838 ± 87 | ND | 316.4 ± 79.5 | | 9.6 ± 1.2 |  |
|  |  | Polyester-cotton jersey | 50% cotton,  50% recycled polyester | None | 103 ± 24 | 351,953 ± 113,603 | 586 ± 141 | | 11 ± 1.3 |  |
|  |  | 18.5 Micron merino wool single jersey | 100% wool | None | 129 ± 18 | 703,023 ± 95,013 | 514 ± 493 | | 11 ± 1.1 |  |
|  | Filament | Heathered sweater fleece | 100% virgin polyester | Napped | 111 ± 32 | 626,894 ± 178,379 | | 313 ± 181 | 12.2 ± 0.6 | Fleece (n=10) |
|  |  | Polyester microfiber fleece (75d/144f) | 100% virgin polyester | Napped | 80 ± 29 | 689,968 ± 254,177 | 256 ± 51 | | 7.8 ± 0.3 |  |
|  |  | Double-sided fleece | 100% virgin polyester | Napped | 167 ± 0.9 | 2,800,356 ± 15,862 | 239 ± 106 | | 11 ± 0.3 |  |
|  |  | Knitted crossdye fleece | 100% virgin polyester | Napped | 127 ± 13 | 348,865 ± 35,418 | 598 ± 541 | | 12 ± 0.4 |  |
|  |  | Two side brushed fleece | 100% virgin polyester | Napped | 80 ± 29 | 1,741,706 ± 625,388 | 324 ± 52 | | 6.8 ± 0.3 |  |
|  |  | Two side brushed fleece with anti-pilling | 100% virgin polyester | Napped | 142 ± 23 | 120,261 ± 19,383 | 653 ± 334 | | 9.5 ± 0.7 |  |
|  |  | Soft double velour recycled fleece | 100% recycled polyester | Napped | 778 ± 304 | 4,315,371 ± 1,683,848 | 528 ± 135 | | 11 ± 0.9 |  |
|  |  | High-pile, shaggy fleece | 70% recycled polyester,  30% virgin polyester | Napped | 269 ± 84 | 530,691 ± 164,884 | 220 ± 198 | | 14 ± 0.7 |  |
|  |  | Knitted crossdye fleece | 100% recycled polyester | Napped | 134 ± 13 | 1,055,497 ± 104,140 | 318 ± 196 | | 11 ± 0.5 |  |
|  |  | Recycled polyester fleece | 64% recycled polyester,  33% virgin polyester, 3% elastane | Napped | 182 ± 29 | 652,195 ± 102,614 | 7,582 ± 1,269 | | 18 ± 0.7 |  |
|  |  | Lightweight knit jersey | 100% virgin polyester | Brushed | 21 ± 4 | 84,288 ± 15,696 | 594 ± 46 | | 14 ± 0.6 | Knit filament jersey with MT (n=5) |
|  |  | Hardface jersey with velour back | 90% virgin polyester, 10% elastane | Brushed or sanded and sheared  ** | 98 ± 37 | 171,985 ± 64,746 | 642 ± 74 | | 14. ± 0.3 |  |
|  |  | Grid-backed jersey | 93% virgin polyester, 7% elastane | Brushed | 60.2 ± 0.4 | 221,446 ± 2,081 | 1,003 ± 78 | | 13 ± 0.7 |  |
|  |  | Sanded single jersey | 93% virgin polyester, 7% elastane | Sanded | 178 ± 109 | 73,915 ± 45,022 | 274 ± 138 | | 9 ± 0.7 |  |
|  |  | Brown high pile jersey | 70% recycled polyester, 30% virgin polyester | Brushed | 370 ± 63 | 87,240 ± 14793 | 434 ± 303 | | 18 ± 1.2 |  |
|  |  | Double dyed heather stretch knit | 46% nylon,  46% Polyester,  8% Spandex | None | 14 ± 4 | 96,194 ± 23,622 | 733 ± 266 | | 9.7 ± 0.7 | Knit filament no MT (n=2) |
|  |  | Mini waffle textured double knit pique | 93% virgin polyester, 7% elastane | None | 49 ± 10 | 568,222 ± 163,358 | 552 ± 125 | | 8.6 ± 0.6 |  |
| Woven | Spun staple | Woven cotton flannel | 100% cotton | None | 215 ± 31 | 648,122 ± 92,390 | 260 ± 153 | | 13 ± 1.7 | Woven spun staple (n=3) |
|  |  | Cotton yarn dyed plaid | 100% cotton | Brushed | 190 ± 22 | ND | 646 ± 111 | | 6.1 ± 0.8 |  |
|  |  | Spun polyester plaid | 100% virgin polyester | Brushed | 174 ± 30 | 592,571 ± 100,928 | 352 ± 336 | | 9.6 ± 1.1 |  |
|  | Filament | Nylon plain weave | 100% virgin nylon | None | 16 ± 2.7 | ND | 517 ± 189 | | 9.3 ± 0.9 | Woven filament (n=8) |
|  |  | Brushed-back soft shell | 92% virgin nylon,  8% elastane | Brushed | 20 ± 11 | 49,594 ± 26,242 | 282 ± 176 | | 10 ± 1.0 |  |
|  |  | Stretch double weave | 88% virgin nylon, 12% elastane | None | 24 ± 5.1 | 20,038 ± 4,342 | 303 ± 399 | | 12 ± 0.5 |  |
|  |  | Nylon stretch woven | 96% virgin nylon,  4% elastane | None | 21 ± 2.4 | 35,415 ± 4,096 | 663 ± 432 | | 9.5 ± 0.5 |  |
|  |  | Double weave stretch woven | 88% virgin nylon,  12% elastane | None | 11 ± 2 | 24,083 ± 4,333 | 291 ± 127 | | 12 ± 1.1 |  |
|  |  | Nylon ripstop taffeta (20dx20d) | 100% virgin nylon | None | 31 ± 0.4 | 33,851 ± 453 | 215 ± 235 | | 13 ± 1.2 |  |
|  |  | Recycled nylon woven ripstop | 100% recycled nylon | Crinkle | 63 ± 27 | 118,655 ± 50,355 | 1,317 ± 416 | | 13 ± 0.7 |  |
|  |  | Recycled polyester Taffeta | 100% recycled polyester | None | 19 ± 8 | 145,549 ± 59,476 | 747 ± 1,178 | | 8.1 ± 0.3 |  |
|  |  | Insulated nylon-polyester composite taffeta | Nylon-polyester composite (percentage not available) | None | 31 ± 6.2 | 50,466 ± 10,244 | 270 ± 42 | | 14 ± 0.5 | Woven filament composite (n=5) |
|  |  | Laminated waterproof breathable membrane | Nylon-ePTFE composite, percentage not available | Laminated | 20 ± 3 | 9,777 ± 1,355 | 744 ± 162 | | 11 ± 1.2 |  |
|  |  | Laminated waterproof breathable membrane with brushed backer | Nylon-ePTFE composite, percentage not available | Brushed | 29 ± 10 | 46,893 ± 15,960 | 785 ± 340 | | 14 ± 0.7 |  |
|  |  | Bonded softshell | 35% nylon,  39% polyester,  16% spandex composite | None | 31 ± 15 | 40,409 ± 19,784 | 296 ± 52 | | 9.2 ± 0.6 |  |
|  |  | 2L Waterproof breathable PU | Polyester-PU composite, percentage not available | None | 23 ± 9 | 241,995 ± 93,780 | 583.0 ± 100 | | 11 ± 0.5 |  |

**S2 Table.** Laundering parameters used in the study and adapted from the AATCC 135-2004 Test Method, 2010 [1].

| **Parameter** | **Setting value** |
| --- | --- |
| Wash water level | 34.1 L |
| Water temperature | 41°C |
| Rinse water level | 34.1 L |
| Soak time | 0 min |
| Wash time | 12 min |
| Agitation cycle | Normal or 120 ± 2 spin per minute  (maximum for the machine) |
| Spin time | 6 min |
| Spin speed | 645 ± 15 rpm |

**S3 Table.** Textile microfiber shedding in the present study and the published literature [2–6].

| **Textile type** | **Present study**  **(mg kg^-1^ per wash)** | **Published study**  **(mg kg^-1^ per wash)** | **Laundering parameters**  **and collection** | **Reference** |
| --- | --- | --- | --- | --- |
| Polyester fleece  (wash number unknown) | *84,000─4,300,000, *n*=14 | **> 3,800, *n*=3 | Front-loading machine  No detergent | Browne *et al.* 2011 |
| Polyester fleece  (1^st^ wash) | 95.2─629, *n*=7 | 161, *n*=1 | Front-loading machine  No detergent  200 μm sampling filter | Pirc *et al*. 2016 |
| Polyester fleece  (5^th^ wash) | 48─567, *n*=9 | 17, *n*=1 |  |  |
| Polyester fleece  (5^th^ wash) | 70,000 to 3,100,000,  *n=*14 | 82,672, *n*=1 | Front-loading machine  With and without detergent  25 μm sampling filter | Napper & Thompson 2016 |
| Polyester-cotton blend,  (5^th^ wash) | 270,000, *n*=1 | 22,992, *n*=1 |  |  |
| Polyester 100%, (2^nd^ & 3^rd^ washes) | ***24─1,183, *n*=13 | ~10, *n*=1 | Top-loading machine  20 μm sampling filter |  |
| Polyester 50% Cotton 50%  (2^nd^ & 3^rd^ washes) | ***100─820, *n*=2 | ~40, *n*=1 |  | Zambrano *et al*. 2019 |
| Cotton 100%,  (2^nd^ & 3^rd^ washes) | ***132─228, *n*=3 | ~60, *n*=1 |  |  |
| Polyester fleece and microfleece  (2^nd^ wash) | 270─24,000  fibers per 100 cm^2^, *n*=7 | 932─1,210  fibers per 100 cm^2^, *n*=3 | Accelerated laundry Detergent  1.2 μm sampling filter | Almroth *et al.* 2018 |
| Knit fabrics  2^nd^ wash | 200─24,000  fibers per 100 cm^2^  *n=*13 | 9 ± 7 fibers  per 100 cm^2^  *n*=7 |  |  |

*An average of three consecutive washes (3^rd^,4^th^ and 5^th^).

**Calculated assuming that a single garment represents 500 g of textile.

*** Averages from consecutive washes.

**S1 Fig**. Custom-built washing machine laboratory (left) and effluent collection manifold (right).

*
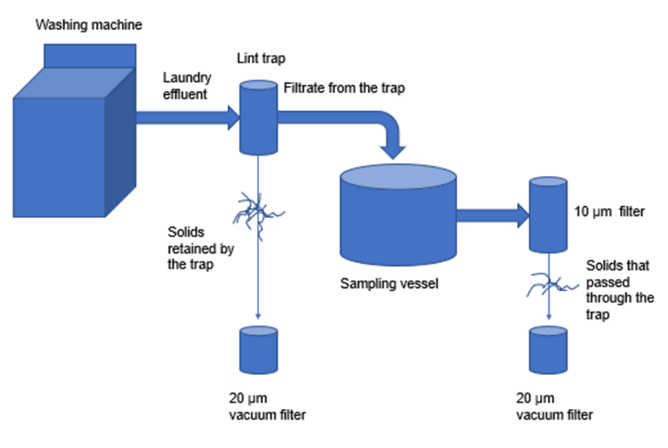
*

**S2 Fig**. A schematic diagram of lint trap evaluation method.


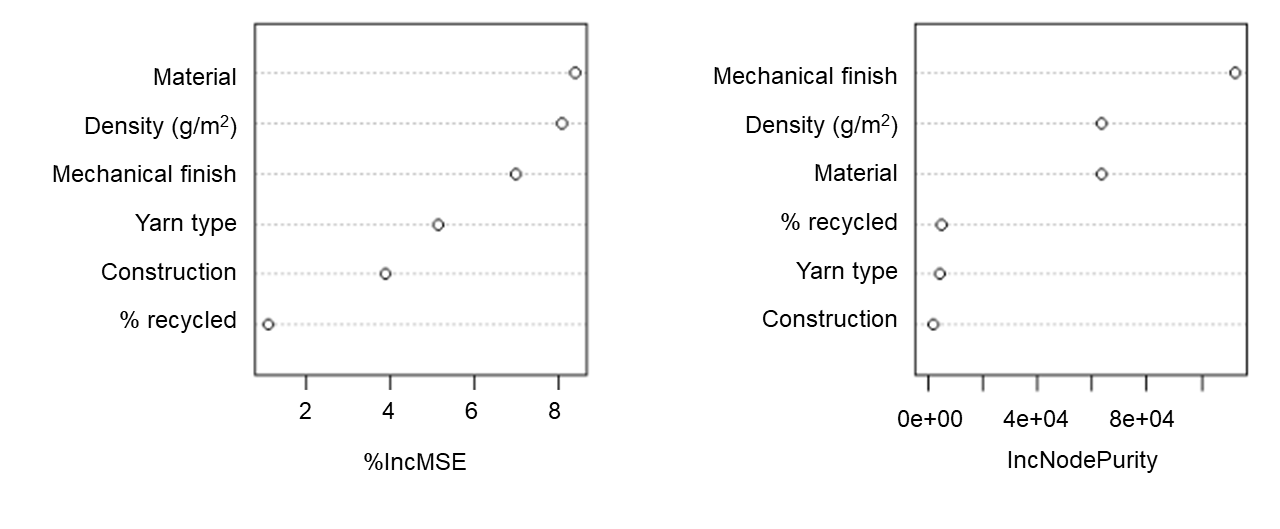


**S3 Fig.** Mean Decrease Accuracy (%IncMSE) and Mean Decrease Gini (IncNodePurity) (sorted decreasingly from top to bottom) of textile design features assigned by the RF model. The model outputs indicate that material type, density and mechanical finish had the strongest influence on fiber release by the 37 materials investigated in this study.


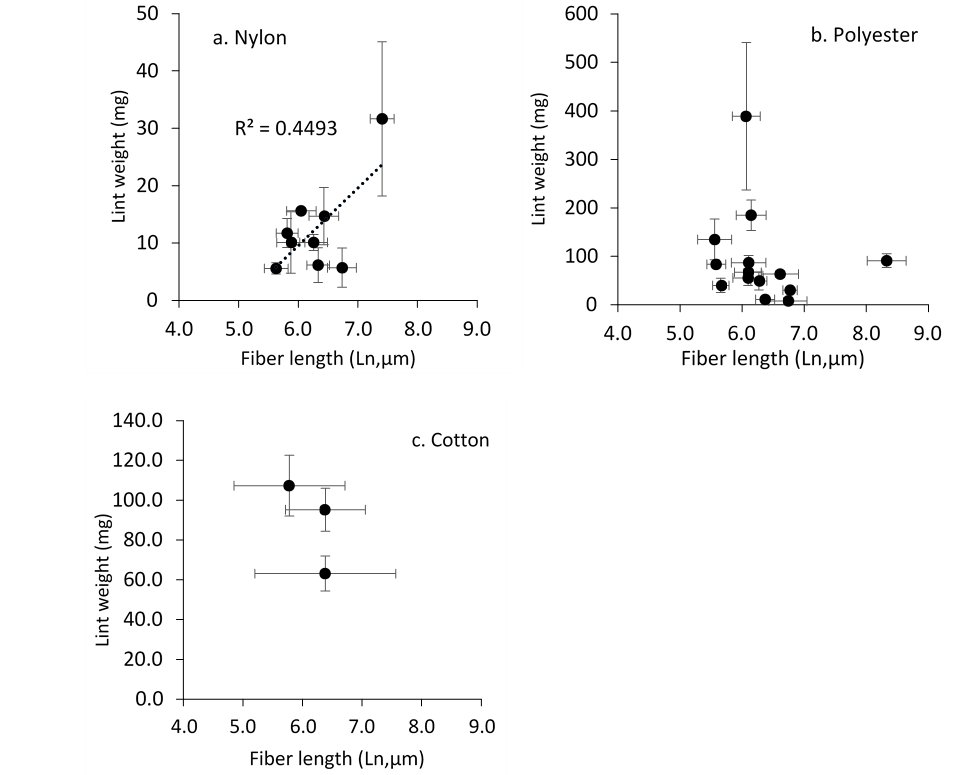


*p* = 2.37e-06

**S4 Fig.** Relationship between the lint weight (mg) and median length of the fibers released in domestic laundry (Ln transformed) for nylon, polyester and cotton material categories. Errors represent standard deviations (SD).

Supplementary Material References

1. AMERICAN ASSOCIATION OF TEXTILE CHEMISTS AND COLORISTS. AATCC Test Method 135-2004. Dimensional changes of fabrics after home laundering. AATCC TECHNICAL MANUAL. 2010. pp. 221–224. Available: www.aatcc.org.

2. Browne MA, Crump P, Niven SJ, Teuten E, Tonkin A, Galloway T, et al. Accumulation of microplastic on shorelines woldwide: Sources and sinks. Environ Sci Technol. 2011;45: 9175–9179. doi:10.1021/es201811s

3. Pirc U, Vidmar M, Mozer A, Kržan A. Emissions of microplastic fibers from microfiber fleece during domestic washing. Environ Sci Pollut Res. 2016;23: 22206–22211. doi:10.1007/s11356-016-7703-0

4. Napper IE, Thompson RC. Release of synthetic microplastic plastic fibres from domestic washing machines: Effects of fabric type and washing conditions. Mar Pollut Bull. 2016;112: 39–45. doi:10.1016/j.marpolbul.2016.09.025

5. Zambrano MC, Pawlak JJ, Daystar J, Ankeny M, Cheng JJ, Venditti RA. Microfibers generated from the laundering of cotton, rayon and polyester based fabrics and their aquatic biodegradation. Mar Pollut Bull. 2019;142: 394–407. doi:10.1016/j.marpolbul.2019.02.062

6. Carney Almroth BM, Åström L, Roslund S, Petersson H, Johansson M, Persson NK. Quantifying shedding of synthetic fibers from textiles; a source of microplastics released into the environment. Environ Sci Pollut Res. 2018;25: 1191–1199. doi:10.1007/s11356-017-0528-7
